# An Observational View of Relationships Between Moisture Aggregation, Cloud, and Radiative Heating Profiles

**DOI:** 10.1007/s10712-017-9443-1

**Published:** 2017-10-31

**Authors:** Matthew D. Lebsock, Tristan S. L’Ecuyer, Robert Pincus

**Affiliations:** 1 Jet Propulsion Laboratory, California Institute of Technology, Pasadena, CA 91109 USA; 2 University of Wisconsin, Madison, WI 53706 USA; 3 Cooperative Institute for Research in Environmental Sciences, University of Colorado, Boulder, CO 80309 USA; 4 Physical Sciences Division, NOAA Earth System Research Lab, Boulder, CO 80305 USA

**Keywords:** Convective aggregation, Radiation, Water vapor, Satellite, Observations

## Abstract

Data from several coincident satellite sensors are analyzed to determine the dependence of cloud and precipitation characteristics of tropical regions on the variance in the water vapor field. Increased vapor variance is associated with decreased high cloud fraction and an enhancement of low-level radiative cooling in dry regions of the domain. The result is found across a range of sea surface temperatures and rain rates. This suggests the possibility of an enhanced low-level circulation feeding the moist convecting areas when vapor variance is large. These findings are consistent with idealized models of self-aggregation, in which the aggregation of convection is maintained by a combination of low-level radiative cooling in dry regions and mid-to-upper-level radiative warming in cloudy regions.

## Introduction

Radiative-convective equilibrium, in which heating of the atmosphere by moist convection and precipitation balances radiative cooling, is an idealization of the Earth’s tropical atmosphere that neglects advective energy transport. Over a uniform surface, and in domains large enough to contain many convective elements, the null hypothesis would be that convection in radiative-convective equilibrium would be distributed roughly uniformly throughout the domain. It has been known for more than 20 years, however, that convection in numerical model simulations of radiative-convective equilibrium frequently gathers itself together, increasing in spatial scale until, under many circumstances, the entire domain contains only a single region of convection (Held et al. 1993; Bretherton et al. 2005; Stephens et al. 2008; Wing and Emanuel 2014; Tompkins and Craig 1998). The phenomenon of convective ‘self-aggregation’ was originally noted in cloud-resolving models in which deep convection is explicit (Held et al. 1993; Bretherton et al. 2005; Stephens et al. 2008; Wing and Emanuel 2014) but it also appears in global models (Bony et al. 2016; Shi and Bretherton 2014; Reed et al. 2015; Coppin and Bony 2015) in which convection is parameterized.

Self-aggregation has primarily been found to occur in simulations with warm Sea Surface Temperatures (SST) with a possible SST threshold below which aggregations does not occur (e.g., Wing and Emanuel 2014). A precise threshold remains elusive: Self-aggregation has been simulated at SSTs less than 300 K (Wing et al. 2016; Holloway and Woolnough 2016) and there are even simulations with an upper SST bound above which self-aggregation does not occur (Wing and Emanuel 2014). While studies have consistently found a relationship between temperature and self-aggregation, there is not a consensus on the specific nature of this relationship. The remainder of this paper will address aggregation in warm SST environments with the caveat that the phenomena may have broader applicability.

Models show that in a fully aggregated state, the atmosphere consists of a few very moist regions containing strong convection and a much larger dry, subsiding region. The contrast in humidity between dry and moist regions is strong so that the variance of the moisture field increases with the degree of aggregation (Bretherton et al. 2005; Wing et al. 2016). High clouds are less frequent in aggregated states, allowing increased longwave (LW) radiative cooling to space. The precise details of convectively aggregated states are still being explored (Muller and Bony 2015) but the frequent finding that aggregation increases with sea surface temperature suggests a stabilizing feedback on climate reminiscent of the ‘Iris hypothesis’ (Lindzen et al. 2001), albeit through different mechanisms than originally proposed (Mauritsen and Stevens 2015; Bony et al. 2016).

The degree to which the self-aggregation of convection is relevant to the Earth’s atmosphere is not entirely clear. As reviewed carefully by Holloway et al. (2017; this issue), the organization of convection has been thought of for many years as being intimately linked to mesoscale systems organized by gravity and other convectively coupled waves (e.g., Mapes 1993) or large-scale circulations. The Earth’s atmosphere does exhibit some characteristics of convective self-aggregation, including the tendency of more organized atmospheres to have lower humidity in clear areas, reduced domain-mean high cloudiness, and increased low cloudiness in non-convective areas, with corresponding impacts on surface and radiative fluxes (Tobin et al. 2012; Stein et al. 2017; Tobin et al. 2013).

As the mechanisms responsible for self-aggregation in radiative-convective equilibrium become more robustly understood, observational tests focusing on those mechanisms become possible (see Holloway et al., this issue; Bony et al., this issue). Here, we expand on the relatively small literature (Tobin et al. 2012, 2013; Stein et al. 2017) examining how the structure of the atmosphere, and the clouds embedded in it, depends on the degree of organization. We exploit a range of colocated observations from the A-Train satellite constellation (Stephens et al. 2002; L’Ecuyer and Jiang 2010) to identify aggregated states in the tropical atmosphere and examine the cloud, precipitation, and radiative structure of these states. We use a new measure of organization based on the spatial variability of the water vapor field. The richness of the observations allows us to identify the circumstances under which aggregation is most frequent and to disentangle the effects of aggregation and mean environmental conditions on the cloud and humidity structure of the atmosphere. We emphasize the important distinction between the observed aggregation that is influenced by external forcing and the idealized concept of self-aggregation, which occurs in the absence of large-scale forcing. The results of this paper must be interpreted with the understanding that these are distinct phenomena.

## Characterizing Aggregation in Clouds and Their Environment

### Observations from the A-Train

We use data products from the A-Train constellation (Stephens et al. 2002; L’Ecuyer and Jiang 2010). These sensors include the Advanced Microwave Scanning Radiometer for EOS (AMSR-E), the Moderate resolution Imaging Spectroradiometer (MODIS), CloudSat, and CALIPSO. AMSR-E data is available from June 2002 to October 2011 when the instrument spun down. MODIS cloud data is used during this same period. CloudSat/CALIPSO data was used from the period June 2006 to February 2011. All the observations are nearly instantaneous snapshots as opposed to daily average quantities. Results that contain CloudSat/CALIPSO data use the 2006–2011 epoch, whereas results that do not use CloudSat/CALIPSO data use the full A-Train period.

Here, we use Column Water Vapor (CWV) from the AMSR-E sensor (Kawanishi et al. 2003) derived from the version 7 Remote Sensing System algorithms (Wentz and Meissner 2000, 2007). The CWV has an expected precision of 1 kgm^−2^. The data product is available over ocean surfaces on a 0.25° daily grid with the ascending orbital nodes separated from the descending orbital nodes.

Surface Rain Rate (RR) data are also derived from the AMSR-E sensor using the version 2 Goddard Profiling (GPROF) algorithm (Kummerow et al. 2011). Like the CWV data, the RR is available on a 0.25° daily grid with the ascending orbital nodes separated from the descending orbital nodes.

Cloud data are taken from the collection 5.1, Level 3 Aqua MODIS products. We use the Cloud top pressure (CTP) histograms in the Level 3 products, which are separately stored for ascending and descending nodes (Cloud_Top_Pressure_Day_Histogram_Counts and Cloud_Top_Pressure_Night_Histogram_Counts). The CTP histograms bin the observed cloud counts as a function of 11 bins in 100 hPa increments from the surface to the top of the atmosphere. In addition to the histogram, we use the CTP counts (Cloud_Top_Pressure_Day_Pixel_Counts and Cloud_Top_Pressure_Night_Pixel_Counts) variable in order to calculate cloud fractions from the histograms.

Cloud occurrence profiles are derived from the release-04 2B-Geoprof-Lidar product (Mace et al. 2009) which combines the CloudSat radar cloud mask (Marchand et al. 2008) with the CALIPSO lidar cloud mask (Vaughan et al. 2009). These data are stored on granules that correspond to single orbits; nadir-only sampling means that there is no overlap between the ascending and descending observations.

Precipitation incidence is used from the release-04 CloudSat 2C-Precip-Column product (Haynes et al. 2009; Smalley et al. 2013). Surface rain incidence is defined using the Precip_flag variable = 3, which corresponds to certain precipitation and a radar reflectivity exceeding 0 dBZ at an altitude of approximately 720 m.

Radiative heating profiles are taken from the CloudSat/CALIPSO 2B-Flxhr-Lidar product (Henderson et al. 2013), which combines meteorological analysis with the cloud and aerosol profile information from CloudSat and CALIPSO and a dynamic land surface as input to a radiative transfer model to compute the profile of radiative fluxes at 240 m vertical resolution. Pixel-level RMS differences between this product and the derived top of the atmosphere (TOA) fluxes from the Clouds and Earth Radiant Energy System (CERES) are 5.7 and 16.5 W m^−2^ for the longwave and shortwave, respectively. However, the biases in pixel-scale retrievals are less than 5 W m^−2^ (Henderson et al. 2013). We use a modified version of the 2B-Flxhr-Lidar product designed to estimate the diurnal mean fluxes. This modified product computes the shortwave fluxes using 12 different solar zenith angles to account for the diurnal precession of the incoming flux. This product does not, however, account for diurnal changes in the cloud or thermodynamic variables, since they are not directly observed by the A-Train constellation. The Flxhr-lidar product includes an estimate of the Cloud Radiative Effect (CRE). The CRE is calculated explicitly by performing the radiative transfer calculation twice: once all sky and once clear sky. The CRE is then calculated as the difference of the clear-sky calculation from the all-sky calculation. All fluxes that follow are defined positive downward.

### Characterizing Aggregation in the Water Vapor Field

Observational studies of convective self-aggregation to date (Tobin et al. 2012; Stein et al. 2017; Tobin et al. 2013) have quantified the degree of aggregation based on the degree to which cold clouds observed with a domain are spatially coherent, and these studies have also noted that clear areas tend to be less humid when convection is more aggregated. We invert this logic using a measure of aggregation defined by the inhomogeneity of the integrated water vapor field. Our motivations are partly practical: Definitions of aggregation based on clouds require processing high volumes of pixel-scale cloud observations, and cloud observations can be sensitive to the details of the observing system including sensor resolution, inherent sensitivity, and algorithmic choices (Pincus et al. 2012) while being subject to much larger high-frequency variability than is vapor. More importantly, it is useful to understand the degree to which variability in water vapor can exist independently of the systematic organization of convection.

We define the degree of aggregation *α* using the coefficient of variation for water vapor calculated from the 0.25° data on a 5° twice-daily (day/night) grid,

1 $$\alpha = \frac{{\sigma_{\text{CWV}} }}{{\overline{\text{CWV}} }}$$

where the overbar represents the spatial mean and *σ* is the standard deviation taken over the grid. All other cloud, precipitation, and radiation data are aggregated to a common twice-daily 5° grid. The choice of a 5° grid is arbitrary; however, the conclusions drawn herein do not change when repeating the analysis at 10°. The separation of ascending and descending nodes is important because it keeps each grid box a semi-instantaneous sample in time. Pixels identified as land are filtered out of the analysis with no imposed threshold on the number of ocean pixels that enter each 5° aggregation. The high cloud fraction is calculated from both the MODIS and CloudSat/CALIPSO data, whereas low cloud fraction is only derived from the CloudSat/CALISPO data. From MODIS, the high cloud fraction is calculated using the cloud fraction in the CTP histograms with CTP lower than 400 hPa. Similarly, from CloudSat the high cloud fraction is calculated as the fraction of cloud cover above 7.5 km.

All 5° regions are filtered for SSTs between 300 and 304 K, which include most of the warmest tropical SSTs. Higher SSTs are infrequent enough that sampling is problematic. The lower SST bound is motivated by modeling studies that suggest that SSTs over 300 K are the relevant regime for self-aggregation. We also note that results are robust down to SSTs of 296 K.

Daily data can occasionally be missing in areas of intense precipitation where high winds affect surface emissivity and large ice water contents cause scattering of the emission signal. These missing data points are disregarded in the calculation of the aggregation. The missing data are most likely the high tail of the CWV distribution and therefore may introduce some systematic bias in the calculation of *α*; however, this influence is somewhat mitigated by the normalization in Eq. 1 as both the standard deviation and mean will be biased low when data are missing.

**Fig. 1:**
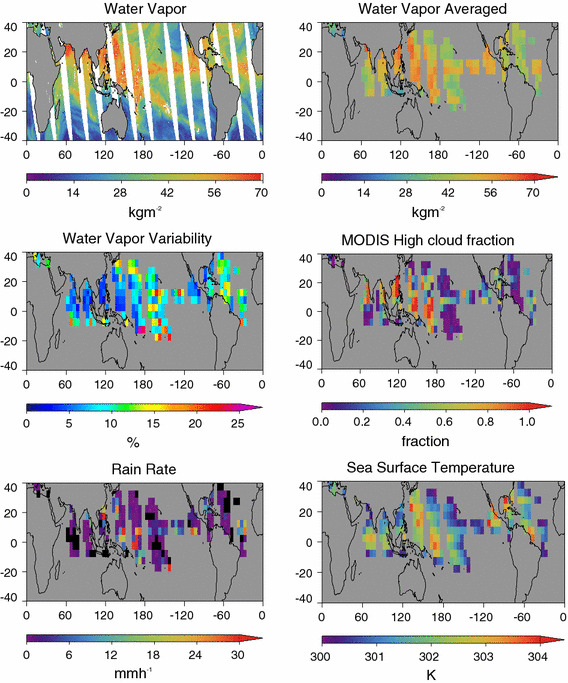
An example of the data for the ascending orbit of August 04, 2006. The top left panel shows the 0.25° native water vapor fields. All other panels show 5° averaged data for pixels with SST between 300 and 304 K

Figure 1 shows an example of 1 day’s calculation of the aggregation and related data products for the ascending (daytime) orbits.

## Relationships Among Clouds, Humidity, and Aggregation

**Fig. 2:**
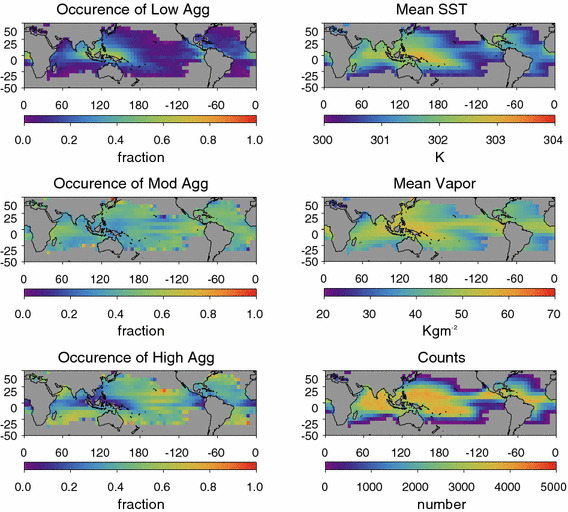
The left-hand panels show the frequency of occurrence of various aggregation states. Low-aggregation is defined as < 5%, moderate-aggregation is defined as 5–10%, and high-aggregation is defined as > 10%. The right-hand panel shows the mean SST, column water vapor, and sample count. Note the predominance of low-aggregation in the maritime continent with areas of higher aggregation on the boundaries of the ITCZ and the warm pool

Our relatively large data set allows us to examine the geographic distribution of aggregation. Figure 2 shows a map of the degree of aggregation, water vapor, and SST over the period 2002–2011. Aggregation is largest where the gradient of the mean water vapor field is largest, on the edges of the West Pacific warm pool and the Inter-Tropical Convergence Zone (ITCZ), suggesting that aggregation, by this measure, is most common in domains which cover both the ascending and descending regions of either a large-scale or a synoptic circulation. This geographical distribution is inconsistent with some modeling results suggesting that aggregation increases with SST although this may be an artifact of the particular definition of aggregation used here.

**Fig. 3:**
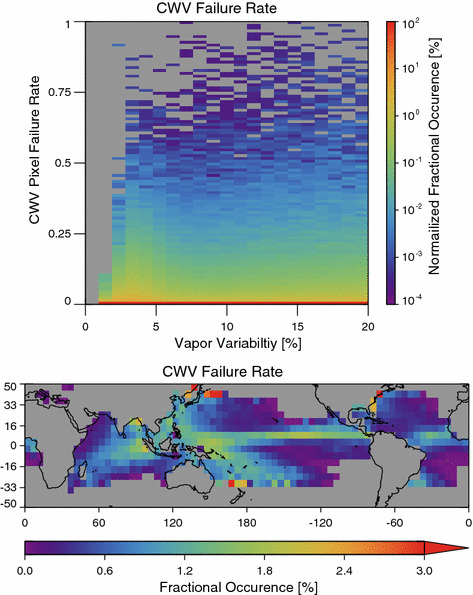
(top) The distribution of Column Water Vapor (CWV) retrieval failure rates as a function of the vapor variability (*α*). Results for each *α* bin are normalized such that the sum for each column adds to 100%. The color scale is logarithmic. Note that for all values of alpha by far the most common occurrence is for a failure rate of 0. (bottom) The geographical distribution of the CWV retrieval failure rate, which clearly shows the imprints of the distribution of precipitation

Missing CWV retrieval failures might introduce systematic bias in the results that follow. However, Fig. 3 shows that these failures are rare and relatively evenly distributed across the range of *α*. There is a modest maximum in the failure count for *α* near 3%. The geographical distribution of failure rate clearly shows that the prevalence of failed CWV retrievals follows the distribution of precipitation with relative maximum in the ITCZ and the warm pool. The fraction of missing pixels only exceeds 2% in a handful of poorly sampled grids. Comparing this distribution to Fig. 2, one cannot find a strong correlation between the occurrences of high, moderate, or low mean aggregation state with the CWV failure rate. For example, while both the ITCZ and the warm pool have relatively elevated precipitation rates and retrieval failure rates, the ITCZ is characterized by large *α* and the warm pool by low *α*.

### Cloudiness Depends on Sea Surface Temperature and Aggregation State

**Fig. 4:**
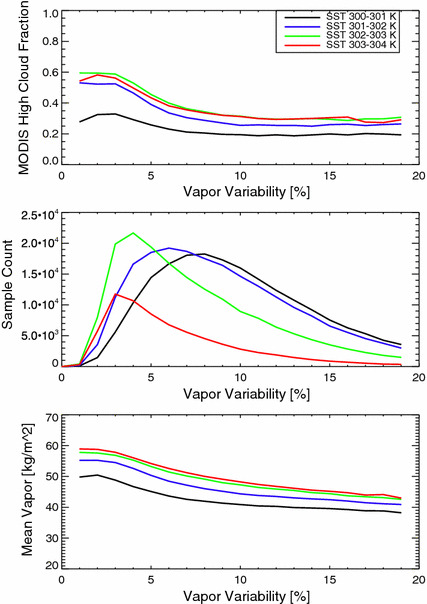
The MODIS high cloud fraction (< 400 hPa), sample count and mean water vapor as a function of the water vapor aggregation

One of the most robust features of self-aggregation in idealized simulations is the reduction in high cloud cover with increased aggregation. This feature appears in our data set, echoing results from previous observational studies. Figure 4 shows that the MODIS high cloud cover tends to decrease with increasing aggregation, and results (not shown) using the CloudSat/CALIPSO data confirm this result. The result is consistent with Stein et al. (2017). That study further finds that the strong dependence of high cloud fraction with aggregation is largely a function of variations in optically thin cirrus cloud. Note that in Fig. 3, panel C shows how the mean water vapor varies with *α*. It is not surprising to see that $\overline{\text{CWV}}$ decreases with *α,* contributing to changes in *α*, since $\overline{\text{CWV}}$ appears in the denominator of Eq. 1, but these changes in the $\overline{\text{CWV}}$ do not explain the majority of the variation in *α*. Therefore, the degree of aggregation is primarily driven by spatial variation in water vapor, not changes in $\overline{\text{CWV}}$. We infer that changes in cloud morphology correlated with *α* are related to changes in the spatial variability of water vapor as opposed to the mean water vapor. We can also observe from Fig. 4 panel C that dry states tend to display a more aggregated state than do moister states which is consistent with the geographical distributions shown in Fig. 2, which shows a minimum aggregation in the moistest regions.

**Fig. 5:**
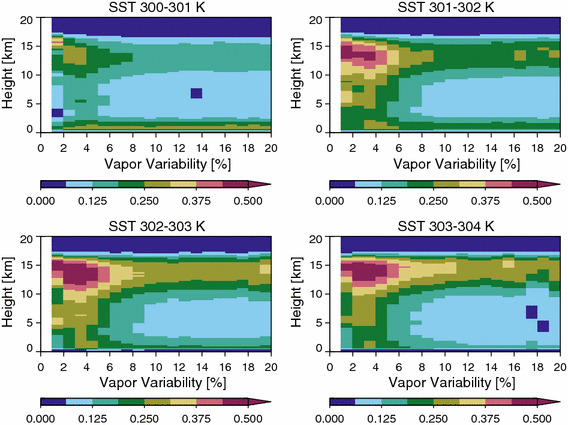
The height resolved cloud fraction from CloudSat/CALIPSO as a function of the aggregation index

Figure 5 shows how the vertical profile of cloudiness changes with increases in *α* (see also Stein et al. 2017, Fig. 3). Profiles are derived from the CloudSat/CALIPSO data which only provide a narrow nadir swath within each sample grid box; however, averaged over a large number of samples, it should provide an unbiased estimate of the mean. This is evident in the fact MODIS and CloudSat/CALIPSO show the same dependence of high clouds on *α*. We see in Fig. 5 that the total cloud cover is a strong function of mean SST but, for a given SST range, an increase in vapor variability is not only associated with a decrease in high cloud fraction but also a decrease in mid-level clouds indicating a drying out of the mid-troposphere, presumably due to a decrease in the convective area fraction. Smaller changes in the low cloud fraction are observed that depend on the SST. At cooler SST, the low cloud fraction increases slightly with *α,* whereas it decreases with *α* at higher SST.

### Cloudiness, Radiative Heating, and Convective Intensity

Figures 1, 2, 3, 4, and 5 mix many different convective states across the aggregation index *α*. Might these results be the result of systematic variation in convective activity with the vapor variance, rather than an indication of the aggregation of convection? To address this concern, we further stratify our results by the observed rain rate averaged over each 5° × 5° region following the approach of Stein et al. (2017). This admittedly rough metric for convective intensity is the best available from the A-Train observations. A better measure of convective area fraction might be gleaned from Global Precipitation Measurement (GPM) mission or Tropical Rainfall Measurement Mission radar observations, which can identify convective precipitation using the spatial variance of the radar reflectivity; however, these observations are rarely coincident with the A-Train data used in this study. The GPM mission includes the GPM Microwave Radiometer (GMI), which has similar characteristics to the AMSR-E radiometer, so it would be possible to examine GPM radar observations in terms of the aggregation index defined in this paper.

**Fig. 6:**
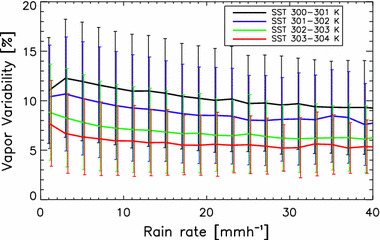
The mean vapor variability (*α*) as a function of mean rain rate within a 5° grid box. The error bars show the standard deviation. While virtually any value of *α* can be observed for any rain rate or SST value, there are clear tendencies for alpha to increase with decreasing rain rate and decreasing SST

Figure 6 shows how the mean *α* depends on both the grid-mean rain rate and SST. There is a tendency toward larger *α* with decreases in either SST or rain rate. Thus, stratifying results by both SST and rain rate is important to determine whether the dependences of clouds on *α* are related to variations in *α* itself or are instead potentially due to correlation of *α* with precipitation.

**Fig. 7:**
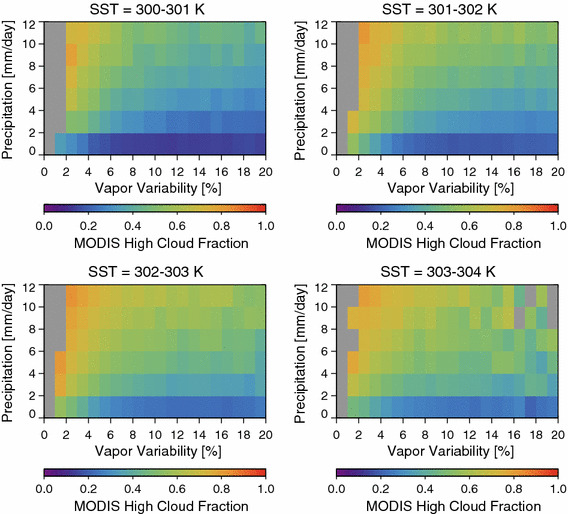
MODIS high cloud fraction as function of the grid box aggregation, mean precipitation rate, and sea surface temperature (SST)

High cloud fraction does indeed decrease with increasing *α* for each rain rate bin and each SST (Fig. 7), with the dependence of high cloud fraction on aggregation similar within each bin. This suggests that the high cloud fraction decreases with *α* due to aggregation of convection, not via a systematic dependence of convective intensity on the water vapor variance.

**Fig. 8:**
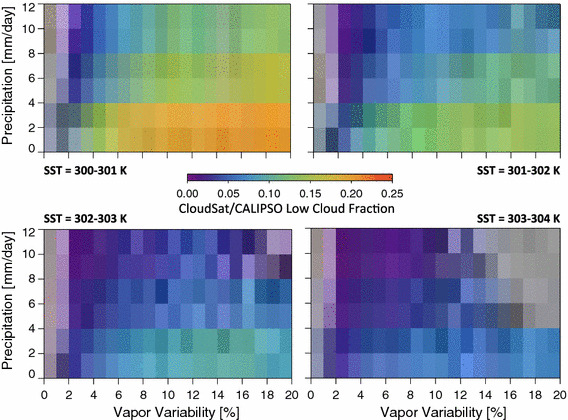
The low cloud fraction as a function of vapor variability, rain rate, and SST. Low clouds are observed using the combined CloudSat/CALIPSO data set and are defined here as clouds having tops lower than 3 km. Low cloud increases with the vapor variability for the majority of rain rate and SST bins

Stein et al. (2017) found that low-level cloud fraction increases along with their aggregation metric. Modeling results also suggest that low-level clouds are crucial for the onset of convective aggregation and are one of the several processes that help maintain an established aggregated state (Muller and Bony 2015). Figure 8 shows that the low cloud fraction as deduced from the CloudSat/CALIPSO data shows increases with alpha, in agreement with the Stein et al.’s (2017) result. Low cloud fraction decreases with increasing SST, decreases with increasing rain rate, and low cloud fraction tends to increase with *α* regardless of the rain rate or SST bin.

**Fig. 9:**
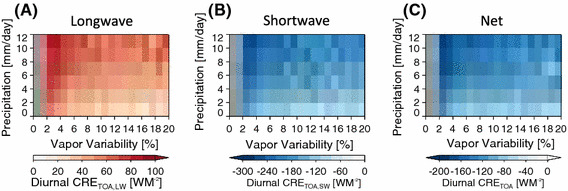
The top of the atmosphere (TOA) longwave (**a**), shortwave (**b**), and net (**c**) cloud radiative effect as function of the grid box aggregation, mean precipitation rate, and sea surface temperature (SST). The convention is positive downward for all fluxes. Fluxes are derived from the diurnally averaged 2B-Geoprof-Lidar product. These results are for SST between 301 and 302 K. Cloud Radiative Effect is the result for other SST’s show a similar dependence on mean precipitation rate and vapor variability

The systematic dependence of the cloud cover on *α* has a substantial influence on the cloud radiative effects. Figure 9 shows the result of the aggregation state on the mean cloud radiative effect at the TOA. As α increases (and high cloud cover decreases), there is increased domain average longwave emission to space compensated by decreased solar reflectance. In general, the shortwave effect is larger than the longwave effect. Results are shown only for the 301–302 K SST bin; qualitatively, similar dependence of the TOA fluxes is found at the other SSTs. For a given rain rate and SST, therefore, net absorption by the earth and atmosphere increases with the degree of aggregation *α.*

**Fig. 10:**
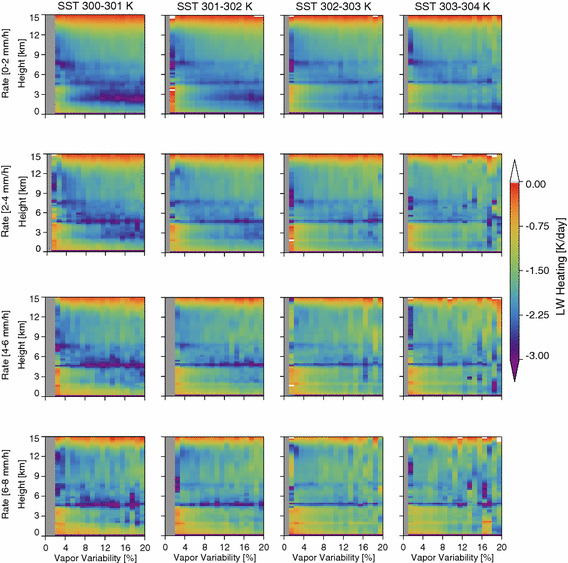
The vertical profile of the CloudSat/CALIPSO diurnal averaged LW heating rate as a function of the vapor variability

The compensation between longwave and shortwave at the TOA implies a redistribution of heating in the atmospheric column with increased solar heating of the surface and increased longwave cooling of the atmosphere. Longwave radiative cooling is concentrated at the effective emission level, which is governed by cloud top and the water vapor scale height. Indeed, heating rate profiles stratified according to rain rate (Fig. 10) show increasing low-level cooling of the atmosphere with increased aggregation. As *α* increases, the height of the maximum cooling decreases and the magnitude of the lower tropospheric cooling increases, each of which supports the LW radiative-convective feedback conjecture whereby enhanced low-level atmospheric cooling with aggregation helps sustain the aggregated state through positive feedback on the regional circulation.

### Does Vapor Aggregation Imply Convective Aggregation?

The results already shown, using a measure of aggregation defined by the water vapor field, show variations of cloudiness consistent with observations stratified by the connectedness of clouds themselves, suggesting that large-scale variance of water vapor and the mesoscale distribution of clouds are tightly linked. In this section, we explore these relationships more carefully.

**Fig. 11:**
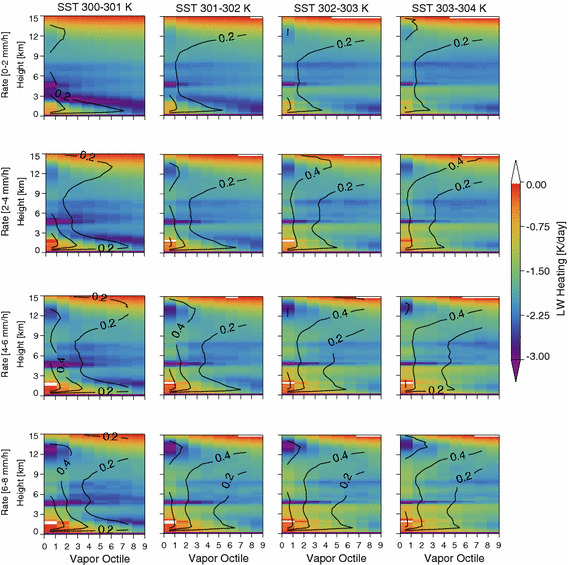
Composite view of the LW cooling rate (colors) and cloud fraction (contours) as a function of vapor octiles averaged over all aggregation states. By definition, the moistest octiles are on the left and the driest octiles on the right side of the plots

We examine differences in the structure of the cloud and radiation fields in the dry and moist areas by compositing our observations as a function of mean column-integrated water vapor. Each twice-daily grid box is divided into water vapor octiles, and then cloud and radiation data are composited as a function of each octile (Fig. 11). This analysis averages data across the various aggregation states while retaining information of the covariability of moisture and cloudiness within each domain. Because SST variation across each 5° box is relatively small, this is analogous to energy-budget analyses used to diagnose the mechanisms leading to self-aggregation (Bretherton et al. 2005; Wing and Emanuel 2014; Muller and Bony 2015) although open boundary conditions suggest that inferring circulations from this stratification is unwise.

Within each rain rate/SST regime, the moistest areas have the highest cloud fraction at all levels, while the driest show very little cloud in the middle atmosphere and enhanced low-level cooling. This picture is consistent with modeling results showing preferential convection in the moist regions that can be maintained (or caused) by an enhanced LW radiative cooling in the dry region (c.f. Figure 2 in Muller and Bony (2015)).

**Fig. 12:**
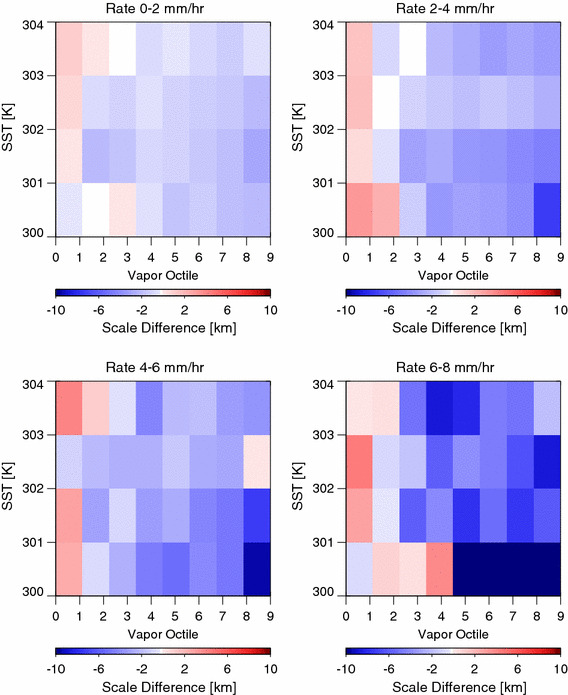
The difference in the precipitation length scale (*l*_*p*_) between vapor variance greater than 10% cases and vapor variance less than 5% cases. In the moistest octile, the aggregated cases tend to have a longer precipitation length scale, whereas the precipitation length scale is shorter in drier octiles

Aggregated convection might also be expected to lead to more aggregated precipitation. The hypothesis is tested using a precipitation length scale *l*_p_, defined as the chord length of contiguous areas of precipitation, based on precise precipitation incidence flags from CloudSat (Smalley and L’Ecuyer 2015). On the scale of an individual sample, this chord length may have a great deal of uncertainty due to the nadir sampling of CloudSat and the non-isotropic structure of precipitation. We make the assumption that averaged over a large number of samples, systematic differences in precipitation spatial scale can be inferred from the chord-length measurement. Figure 12 contrasts *l*_*p*_ in regions with very aggregated regions (*α* > 10) with homogenous regions (*α* < 5). Precipitation length scale is generally longer in the moistest octile for the *α* > 10 state than for *α* < 5, whereas it tends to be shorter in the other 7 octiles, regardless of SST or rain rate. This would occur if, for example, rain from convective systems increasingly aggregates in moist regions as water vapor variance increases. Commensurate with these changes in the character of precipitation in the moist region is a decrease in the organization in the dry areas, which may have more isolated shallow convection.

## Summary and Discussion

We have explored the relationships among clouds, precipitation, radiation, and a measure of the convective aggregation given by the variance in the column water vapor field in large domains over the tropical oceans. This study was motivated by a number of modeling studies hypothesizing an increase in convective aggregation with warming SSTs. Cloud modeling studies of this convective self-aggregation robustly find that the water vapor variance increases with aggregation of the convection. Over ocean surfaces, column water vapor is well-measured and relatively continuous. We expect it provides a measure complementary to the infrared cloud observations that have been employed by previous observational studies of convective aggregation (Tobin et al. 2012; Holloway, this issue).

In the observations presented here, we see a reduction in the area of high cloud cover and an associated increase in the longwave cooling of the atmospheric column to space, as the degree of aggregation increases. We further observe an increase in the low cloud cover with increased aggregation. The enhanced cooling occurs in the dry regions of the domain, reinforcing the moist-static energy gradient between moist and dry regions. Modeling studies suggest that this radiative effect acts as a positive feedback contributing with other processes to maintain the organization of convection in moist areas (Muller and Bony 2015). It is important here to draw a distinction between the initiation and maintenance of the aggregated state. The Muller and Bony study finds that cooling rates localized at cloud top on the order of ~ 13 K/day are required for the initiation, whereas broad lower tropospheric cooling on the order of ~ 2 K/day is helpful but not necessary for maintaining aggregation. The observations shown here are on the order of the ~ 2 K/day helpful for the maintenance of the aggregated state.

This picture of convective organization is consistent across a range of SSTs and rain rates, which we take as a loose proxy for convective intensity. Sorting the results by rain rate provides some relevance to the cloud-climate-feedback problem in the context of radiative-convective equilibrium. In particular, the results show that regions with very different cloud morphology and associated radiative effects can produce the same mean rain rate. It follows that if the aggregated state becomes more prevalent as SST warms, the Earth system may be able to produce the required rainfall to balance the radiative cooling of the atmosphere while having a significantly reduced amount of high cloud.

An important point not addressed by this study is the issue of the spatial scale over which convection might be expected to aggregate as the climate warms. Will the aggregation tend to occur on the mesoscale, the global scale, or at some scale in between? The cyclical boundary conditions and constraint of mass continuity may mean that modeling studies of self-aggregation are more relevant to global-scale circulations than to the mesoscale. This study supports the view that aggregated convection on the synoptic scale produces an environment with a bimodal moisture distribution including dry regions that produce a positive radiative cooling feedback on the convective circulations. These reinforcing radiative feedbacks on convection have also been noted in interannual variability as manifested in the El Nino Southern Oscillation (Rädel et al. 2016) and are implicit in the global-scale narrowing of the inter-tropical convergence zone (Wodzicki and Rapp 2016).

We emphasize that this study cannot confirm observationally that convection does indeed self-aggregate; testing this hypothesis mechanistically will require targeted observations and analysis (Holloway et al., this issue; Bony et al., this issue). Our survey does demonstrate that certain features of the cloud morphology present in model simulations of self-aggregation are also present in observations of the routine aggregation found in the tropical atmosphere. This suggests that aggregation of convection in Earth’s atmosphere, whatever the mechanism, provides a useful conceptual model through which to view important aspects of cloud feedbacks on climate (Mapes 2016).
